# Associations of living alone and loneliness with neuropsychiatric symptoms in individuals with mild cognitive impairment: A retrospective cross‐sectional study

**DOI:** 10.1002/pcn5.70299

**Published:** 2026-02-10

**Authors:** Kayo Takeda, Hideki Kanemoto, Takashi Suehiro, Yuto Satake, Daiki Taomoto, Shigeki Katakami, Kenji Yoshiyama, Manabu Ikeda

**Affiliations:** ^1^ Department of Psychiatry The University of Osaka Graduate School of Medicine Suita Osaka Japan; ^2^ Health and Counseling Center The University of Osaka Toyonaka Osaka Japan

**Keywords:** living alone, loneliness, mild cognitive impairment, neuropsychiatric symptoms

## Abstract

**Aim:**

Living alone and loneliness are recognized risk factors for neuropsychiatric symptoms in older adults. However, the distinct of the objective condition of living alone and subjective loneliness on specific neuropsychiatric symptoms in individuals with mild cognitive impairment (MCI) remains unclear.

**Methods:**

In this retrospective study, we analyzed data from 312 older adults with MCI (74 living alone and 238 living with family) who attended a university psychiatry clinic. Neuropsychiatric symptoms and subjective loneliness were assessed using the Neuropsychiatric Inventory and the UCLA Loneliness Scale, respectively. Living arrangement (living alone vs. living with family) was recorded. Negative binomial regression with a log link function examined associations of depression, anxiety, delusions, and hallucinations with living alone and loneliness, adjusting for potential confounders. Furthermore, sensitivity analyses were performed by excluding variables with highly unbalanced distributions.

**Results:**

Patients living alone showed significantly higher delusions, hallucinations, and disinhibition scores than those living with family, whereas apathy scores were significantly lower. Multivariate analyses showed that loneliness, but not living alone, was significantly associated with depression and anxiety. Delusions were significantly associated with both loneliness and living alone. In contrast, no significant associations were found between hallucinations and either living alone or loneliness. The significant associations for all neuropsychiatric symptoms were maintained in the sensitivity analysis, except for hallucinations.

**Conclusion:**

Neuropsychiatric symptoms in individuals with MCI show differential associations with loneliness and living alone. These findings suggest that useful non‐pharmacological interventions may differ depending on the symptoms.

## INTRODUCTION

With increasing life expectancy, the proportion of older adults in the global population is rising rapidly. Individuals aged 65 and older represent 29.1% in Japan, 24.5% in Italy, and 16.8% in North America.^1^ Consequently, the health and welfare of older people have become significant public health challenges globally, with dementia being one of the major topics. In Japan, a nationwide study reported a dementia prevalence of 12.3%, with aging identified as the primary risk factor.^2^ Mild cognitive impairment (MCI), a prodromal stage of dementia, is of particular importance, with a prevalence of 15.5% among older adults in Japan.^2^ The recent development of anti‐amyloid antibody therapy^3^, ^4^ has further emphasized the importance of early diagnosis and interventions at the MCI stage, before progression to overt dementia.

MCI is associated not only with cognitive impairment but also with neuropsychiatric symptoms. Patients with MCI are reported to exhibit high frequencies of depression, apathy, anxiety, and irritability, affecting 35%–85% of individuals.^5^ They also exhibit psychotic symptoms at clinically relevant rates, with delusions reported in 3.8% and hallucinations in 1.8%.^6^, ^7^ To identify older adults with neuropsychiatric symptoms who are at high risk of conversion to dementia, the concept of mild behavioral impairment (MBI)^8^ has been introduced. All five neuropsychiatric symptom domains of the MBI are associated with progression to dementia, with psychosis associated with the highest risk.^9^ Similarly, psychiatric‐onset dementia with Lewy bodies (DLB) has been proposed to describe cases predominant in the prodromal phase.^10^ It is important to address neuropsychiatric symptoms during the stage of MCI.

In Japan, social changes, such as the rise of nuclear families and sex differences in life expectancy, have led to an increase in single‐person households among older adults. Currently, 15.0% of men and 22.1% of women aged 65 and older live alone.^1^ In general, older adults have been reported to show associations between living alone and psychiatric symptoms such as depression, anxiety, and delusions.^11^, ^12^, ^13^ There have also been reports that depression is elevated among older adults living alone, and that higher levels of depression are associated with a more rapid decline in cognitive function.^14^ Living alone is associated with loneliness, reported by 12%–38% of older individuals.^15^ Loneliness is similarly associated with depression, anxiety, somatic symptoms, and hallucinations.^16^, ^17^, ^18^ Conversely, a previous study has suggested that subjective loneliness, rather than the mere condition of living alone, is more strongly associated with the risk of delusions and other neuropsychiatric symptoms.^19^ Therefore, it is important to clarify the relationship between living alone and psychiatric symptoms, including loneliness at the stage of MCI. However, few studies have examined the associations between living alone or loneliness and neuropsychiatric symptoms.

The present study focused specifically on patients with MCI living alone in a Japanese outpatient with dementia, aiming to describe their neuropsychiatric symptom profiles in comparison with those living with family. In addition, the aim of the current study was to examine (1) differences in neuropsychiatric symptoms and loneliness between MCI patient living alone and those who live with family, and (2) the relationships between affective symptoms (depression and anxiety) and psychotic symptoms (delusions and hallucinations) with living alone or loneliness, while accounting for potential confounders such as age, sex, cognitive function, education, and hearing/visual impairment.

## METHODS

### Study design

This retrospective observational study was conducted without any interventional procedures. Patient data were anonymized using unique research identification numbers to ensure privacy. The study adhered to the Declaration of Helsinki (1975, revised in 2008) and received approval from the Research Ethical Committee of the University of Osaka Hospital (Suita, Japan, approval number 19117).

### Diagnosis procedure

At the neuropsychology clinic of the department of psychiatry in the University of Osaka Hospital, all patients referred for suspected neurocognitive disorders underwent standardized diagnostic evaluations by a multidisciplinary team, including psychiatrists, clinical psychologists, occupational therapists, and speech therapists. The assessments incorporated medical history, neuropsychological testing, and caregiver interviews, supplemented by neuroimaging and laboratory investigations as necessary. For patients living alone, if possible, information was obtained from the primary caregiver who maintained contact with the patient on a weekly basis or more frequently. In case where a neurodegenerative disorder was suspected, a standardized battery of assessments was administered, which includes demographic data, neurological and neuropsychological examinations, assessments of neuropsychiatric symptoms and activities of daily living (ADL), as well as measures of dementia severity, caregiver burden, and loneliness.

### Data collection

Data of patients with MCI were identified from the neuropsychology clinic database of the department of psychiatry in the University of Osaka Hospital based on the following criteria: (1) patients visited between August 1, 2020, and March 31, 2024, and underwent evaluations for suspected neurocognitive disorders; (2) 60 years or older; and (3) a Clinical Dementia Rating (CDR) score of 0.5. Cases with missing data on key variables (e.g., Neuropsychiatric Inventory [NPI]‐plus, Mini‐Mental State Examination [MMSE], years of education, whether there are any hearing/visual impairments that may affect the evaluation, mobility assessed by Physical Self‐Maintenance Scale [PSMS], the University of California, Los Angeles‐Loneliness Scale [UCLA‐LS], and cohabitation status) or severe physical impairments affecting testing were excluded. Patients residing in long‐term care facilities were also excluded.

### Clinical variables

The following variables were extracted from the database; age, sex, age at onset, education, medical history, marital history, hearing or visual impairment, history of psychiatric care, and use of psychotropic medications as demographic data; the MMSE and the Addenbrooke's Cognitive Examination‐III (ACE‐III) for cognition; the CDR for dementia severity; the NPI‐plus for neuropsychiatric symptoms; the PSMS and the Instrumental Activities of Daily Living Scale (IADL) for ADL; and the UCLA‐LS for loneliness. In this study, ever‐married status for marital history was defined as having ever been married, including divorce or widowhood. Hearing or visual impairment was identified based on clinical observations by cognitive function assessors, who evaluated whether the impairment was significant enough to interfere with the assessment process, rather than through quantitative audiometric measurements. The history of psychiatric care was defined as the presence or absence of psychiatric consultation before the age of 40. Our definition of “history of psychiatric care” as onset before age 40 is based on the international consensus that psychiatric symptoms emerging after age 40, and especially after age 60,^20^ represent distinct clinical entities that may be linked to neurodegenerative processes. Use of psychotropic medications was defined as the current use of any of the following: antipsychotics, antidepressants, mood stabilizers, anxiolytics, or hypnotics.

The CDR assessed six domains: memory, orientation, judgment and problem‐solving, community involvement, home and hobbies, and personal care. Ratings were based on interviews with patients and their family members, and scores were calculated according to established guidelines. Since this study focused on MCI, the CDR score for all patients was 0.5. The NPI‐plus^21^ is an extended version of the original NPI^22^ that includes an additional subitem for cognitive fluctuation. The NPI^22^ was evaluated in 12 domains, including delusions, hallucinations, agitation, depression, anxiety, euphoria, apathy, disinhibition, irritability, aberrant motor behavior, disturbances, and appetite and eating behaviors, based on information obtained from reliable informants. Each item was scored for severity, frequency, and caregiver distress. In this study, we used composite scores for each item by multiplying frequency by severity.

The PSMS/IADL measures six basic ADL (toilet, feeding, dressing, grooming, physical ambulation, and bathing) and eight instrumental ADL (ability to use telephone, shopping, food preparation, housekeeping, laundry, mode of transportation, responsibility for own medication, and handle finances).^23^, ^24^ Each item is scored as either 0 indicating dependent (assistance required), or 1, indicating independent (no assistance required).

Loneliness was assessed using the UCLA‐LS. Patients rated each item on a 4‐point Likert scale, ranging from “never” to “always” to indicate how well each statement applied to them. Total scores ranged from 20 to 80, with higher scores denoting increased levels of loneliness.

### Statistical analyses

To examine the relationships between neuropsychiatric symptoms and living alone in patients with MCI, differences in the clinical variables were evaluated between those living alone and those living with family using the Mann–Whitney *U* test for continuous and ordinal or continuous variables and the chi‐square test for nominal variables. Forty‐four multiple comparisons were corrected using the Benjamini–Hochberg method.

NBR with a log link function was used to examine the associations of living alone and loneliness with affective (depression and anxiety) and psychotic (delusions and hallucinations) symptoms. The models were adjusting for potential confounders (age, sex, MMSE scores, years of education, hearing or visual impairments, use of psychotropic medications, mobility assessed by PSMS, and marital history). We also conducted sensitivity analysis by excluding variables that exhibited highly unbalanced distributions (hearing impairment and marital status) to ensure the robustness of our primary findings.

These analyses were performed using IBM spss Statistics version 28.0.1.0 (142) (IBM Corp., Armonk, NY, USA), with statistical significance set at two‐tailed *p* < 0.05.

## RESULTS

### Characteristics of patients with MCI living alone compared with those living with family

**Figure 1:**
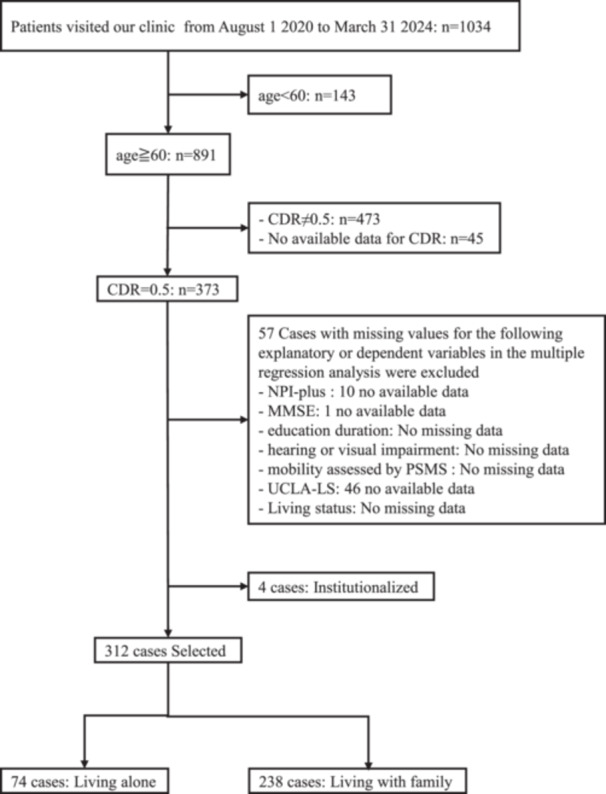
Participant selection flowchart. Abbreviations: CDR, Clinical Dementia Rating; MMSE, Mini‐Mental State Examination; NPI, Neuropsychiatric Inventory; PSMS, the Physical Self‐Maintenance Scale; UCLA‐LS, the University of California, Los Angeles‐Loneliness Scale.

Of data from 1034 patients who visited the hospital between August 1, 2020, and March 31, 2024, 891 were aged 60 years or older, and 373 patients had a CDR score of 0.5. After exclusions, 312 patients were analyzed (74 living alone, 238 living with family) (Figure 1). As shown in Table 1, the living‐alone group had a higher proportion of women, older age, fewer years of education, and a lower rate of ever‐married status. While there were no significant differences in MMSE scores, ACE‐III total, attention, and language score was significantly lower in the living‐alone group than in the living‐with‐family group.

**Table 1:** Clinical characteristics of study participants.

	Living alone (*n* = 74)		Living with family (*n* = 238)		*p*
	Median [IQR]	Mean (SD)	Median [IQR]	Mean (SD)	
Female, *n* (%)	66 (89.2%)		122 (51.3%)		**<0.001**
Age, years	79 [74–83]	78.1 (6.7)	76 [70–80.25]	75.4 (6.8)	**0.003**
Education duration, years	12 [11–14]	12.0 (2.3)	12 [12–16]	13.4 (2.8)	**<0.001**
Ever‐married status, *n* (%)	67 (90.5%)		238 (100.0%)		**<0.001**
Hearing impairment, *n* (%)	10 (13.5%)		16 (6.7%)		0.065
Visual impairment, *n* (%)	0 (0.0%)		2 (0.8%)		0.429
History of psychiatry care, *n* (%)	2 (2.7%)		6 (2.5%)		0.931
Use of psychotropic medications	35 (47.3%)		96 (40.3%)		0.289
MMSE	25 [23–27]	25.2 (2.7)	27 [24–28]	25.7 (3.1)	0.044
ACE‐III	
Total	78 [69.5–86]	76.2 (12.4)	82.5 [76–89]	81.1 (10.2)	**0.003**
Attention	16 [14–17]	15.2 (2.2)	17 [15–18]	16.0 (2.1)	**0.002**
Memory	15 [10–20]	15.2 (5.5)	17 [12.25–22]	16.9 (5.4)	0.025
Fluency	9 [6–10]	8.1 (2.9)	9 [7–11]	8.8 (2.6)	0.070
Language	25 [22–26]	23.3 (3.9)	25 [24–26]	24.5 (2.4)	**0.006**
Visuospatial	15 [14–16]	14.4 (2.1)	15 [14–16]	14.8 (1.8)	0.227
PSMS (independent), *n* (%)	
Toilet	71 (95.9%)		228 (95.8%)		0.956
Feeding	73 (98.6%)		234 (98.3%)		0.844
Dressing	73 (98.6%)		230 (96.6%)		0.367
Grooming	71 (95.9%)		228 (95.8%)		0.956
Physical ambulation	56 (75.7%)		189 (79.4%)		0.505
Bathing	71 (95.9%)		225 (94.1%)		0.545
IADL (independent), *n* (%)	
Ability to use telephone	73 (98.6%)		236 (99.2%)		0.694
Shopping	66 (89.2%)		191 (80.3%)		0.078
Food preparation	49 (74.2%)		91 (74.6%)		0.958
Housekeeping	66 (100.0%)		120 (98.4%)		0.296
Laundry	66 (100.0%)		116 (95.1%)		0.067
Mode of transportation	74 (100.0%)		237 (99.6%)		0.577
Responsibility for own medications	46 (62.2%)		172 (72.3%)		0.098
Ability to handle finances	72 (97.3%)		235 (98.7%)		0.388
NPI	
Delusions	0 [0–6.5]	3.2 (4.7)	0 [0–0]	0.8 (2.5)	**<0.001**
Hallucinations	0 [0–2]	2.0 (4.0)	0 [0–0]	0.8 (2.5)	**0.003**
Agitation	0 [0–0]	0.9 (2.0)	0 [0–0]	0.8 (1.9)	0.814
Depression	0 [0–2]	1.5 (2.7)	0 [0–1]	1.0 (2.2)	0.193
Anxiety	0 [0–2]	1.7 (3.4)	0 [0–1]	1.0 (2.2)	0.169
Euphoria	0 [0–0]	0.0 (0.0)	0 [0–0]	0.0 (0.0)	1
Apathy	0 [0–4]	2.4 (3.0)	4 [0–4]	3.4 (3.1)	**0.005**
Disinhibition	0 [0–0]	0.6 (1.5)	0 [0–0]	0.3 (1.4)	**<0.001**
Irritability	0 [0–0]	0.7 (1.7)	0 [0–0]	0.9 (2.2)	0.806
AMB	0 [0–0]	0.3 (1.7)	0 [0–0]	0.4 (1.7)	0.248
Sleep	0 [0–3]	1.7 (2.7)	0 [0–4]	2.0 (2.8)	0.386
Appetite	0 [0–0]	1.3 (2.8)	0 [0–4]	1.9 (3.3)	0.240
Fluctuation	0 [0–0]	0.8 (2.1)	0 [0–0]	0.7 (1.9)	0.654
Total score	12 [4–26]	16.4 (15.1)	10 [4–18]	13.1 (13.1)	0.141
UCLA‐LS	39 [31–48]	39.8 (10.7)	37 [30–45]	37.9 (10.0)	0.231

*Note*: This table presents the results of the comparison between patients with mild cognitive impairment (MCI) living alone and those living with family. Participants with any missing data for the ACE‐III (total or subscale) were excluded from the comparison analysis. This applied to 1 participant in the living‐alone group and 18 participants in the living‐with‐family group. Data are presented as median (IQR) because non‐parametric tests were used. Mean (SD) is also provided for some variables to better characterize the distribution. Bold values indicate *p* < 0.05 after adjusting for multiple comparisons using the Benjamini–Hochberg method.

Abbreviations: ACE‐III, Addenbrooke's Cognitive Examination‐III; AMB, aberrant motor behavior; IADL, the Instrumental Activities of Daily Living Scale; IQR, interquartile range; MMSE, Mini‐Mental State Examination; NPI, Neuropsychiatric Inventory; PSMS, the Physical Self‐Maintenance Scale; SD, standard deviation; UCLA‐LS, the University of California, Los Angeles‐Loneliness Scale.

Regarding neuropsychiatric symptoms, patients living alone showed significantly higher NPI‐plus subscale scores for delusions, hallucinations, and disinhibition. In contrast, the NPI subscale score for apathy was significantly lower in the living‐alone group. No significant differences were observed in UCLA‐LS scores between the two groups.

### Impact of living alone and loneliness on neuropsychiatric symptoms

Table 2 summarizes the NBR models with a log link function. Regarding depression, the multivariate analysis showed that living alone was not significantly associated with symptom severity, whereas higher loneliness scores were significantly associated with depression. Other factors demonstrating significant associations in the multivariate model included female sex, younger age, ever‐married status, and the use of psychotropic medication. For anxiety, higher loneliness scores remained significantly associated with symptom severity after multivariate adjustment, while living alone showed no significant association. Additionally, significant associations were preserved for female sex, younger age, and the use of psychotropic medications. Regarding delusions, both living alone and higher loneliness scores were consistently associated with symptom severity in the multivariate analysis. Among other covariates, female sex and older age also remained significantly associated with delusions in the multivariate model. Finally, for hallucinations, neither living alone nor loneliness scores showed a significant association in the multivariate model. However, significant associations remained for female sex, hearing impairment, and the use of psychotropic medications. The results of the sensitivity analysis for most neuropsychiatric symptoms (depression, anxiety, and delusions) were generally consistent with our original findings. For hallucinations, the sensitivity analysis revealed a statistically significant association with living arrangement (Table S1).

**Table 2:** Negative binomial regression analysis of Neuropsychiatric Inventory (NPI) subscale scores for depression, anxiety, delusions, and hallucinations.

	Univariate analysis			Multivariate analysis		
	*B* (SE)	IRR [95% CI]	*p*	*B* (SE)	IRR [95% CI]	*p*
NPI depression subscale score						
Female	0.48 (0.17)	1.61 [1.17, 2.23]	**0.004**	1.01 (0.22)	2.74 [1.79, 4.20]	**<0.001**
Age	−0.02 (0.01)	0.98 [0.96, 1.00]	**0.049**	−0.04 (0.01)	0.96 [0.93, 0.99]	**0.004**
Education duration	0.06 (0.03)	1.06 [1.01, 1.12]	**0.027**	0.01 (0.03)	1.01 [0.95, 1.06]	0.843
Ever‐married status	−0.25 (0.50)	0.78 [0.29, 2.06]	0.611	0.17 (0.03)	1.18 [1.10, 1.26]	**<0.001**
Hearing impairment	0.14 (0.27)	1.15 [0.67, 1.97]	0.606	0.38 (0.33)	1.47 [0.77, 2.78]	0.239
Use of psychotropic medications	0.90 (0.16)	2.46 [1.80, 3.37]	**<0.001**	0.70 (0.18)	2.02 [1.43, 2.86]	**<0.001**
MMSE	0.01 (0.02)	0.58 [1.01, 0.97]	0.584	0.01 (0.22)	1.01 [0.66, 1.55]	0.969
Mobility dependence	−0.06 (0.19)	0.94 [0.64, 1.36]	0.735	0.21 (0.57)	1.23 [0.41, 3.76]	0.711
Living alone	0.37 (0.18)	1.45 [1.02, 2.05]	**0.036**	0.17 (0.21)	1.19 [0.78, 1.80]	0.420
UCLA‐LS	0.04 (0.01)	1.04 [1.03, 1.06]	**<0.001**	0.05 (0.01)	1.05 [1.03, 1.07]	**<0.001**
NPI anxiety subscale score						
Female	0.53 (0.16)	1.70 [1.23, 2.34]	**0.001**	0.45 (0.20)	1.56 [1.04, 2.33]	**0.030**
Age	−0.01 (0.01)	0.99 [0.97, 1.01]	0.213	−0.03 (0.01)	0.97 [0.94, 0.99]	**0.019**
Education duration	−0.09 (0.03)	0.91 [0.87, 0.97]	**0.001**	−0.02 (0.03)	0.98 [0.92, 1.05]	0.570
Ever‐married status	0.51 (0.59)	1.67 [0.52, 5.30]	0.387	1.17 (0.63)	3.23 [0.93, 11.13]	0.064
Hearing impairment	0.40 (0.26)	1.49 [0.90, 2.49]	0.123	0.25 (0.30)	1.28 [0.72, 2.28]	0.405
Use of psychotropic medications	0.88 (0.16)	2.41 [1.77, 3.29]	**<0.001**	0.76 (0.17)	2.14 [1.53, 2.99]	**<0.001**
MMSE	−0.02 (0.02)	0.98 [0.93, 1.02]	0.341	−0.01 (0.03)	0.99 [0.94, 1.04]	0.626
Mobility dependence	−0.05 (0.19)	0.95 [0.66, 1.38]	0.793	−0.20 (0.21)	0.82 [0.54, 1.25]	0.362
Living alone	0.55 (0.17)	1.74 [1.24, 2.43]	**0.001**	0.31 (0.20)	1.36 [0.91, 2.03]	0.129
UCLA‐LS	0.03 (0.01)	1.03 [1.01, 1.05]	**<0.001**	0.02 (0.01)	1.02 [1.01, 1.04]	**0.010**
NPI delusions subscale score						
Female	1.35 (0.18)	3.87 [2.72, 5.51]	**<0.001**	1.09 (0.21)	2.99 [1.96, 4.55]	**<0.001**
Age	0.06 (0.01)	1.06 [1.04, 1.08]	**<0.001**	0.03 (0.01)	1.03 [1.00, 1.06]	**0.040**
Education duration	−0.20 (0.03)	0.82 [0.77, 0.87]	**<0.001**	−0.06 (0.04)	0.94 [0.88, 1.01]	0.094
Ever‐married status	−0.23 (0.48)	0.79 [0.31, 2.03]	0.628	−0.39 (0.55)	0.68 [0.23, 2.01]	0.487
Hearing impairment	1.04 (0.24)	2.82 [1.77, 4.50]	**<0.001**	0.32 (0.30)	1.37 [0.76, 2.48]	0.297
Use of psychotropic medications	0.55 (0.15)	1.73 [1.29, 2.33]	**<0.001**	0.24 (0.17)	1.27 [0.90, 1.78]	0.176
MMSE	−0.03 (0.03)	0.97 [0.93, 1.02]	0.310	0.01 (0.03)	1.01 [0.95, 1.06]	0.797
Mobility dependence	0.61 (0.17)	1.85 [1.32, 2.59]	**<0.001**	0.26 (0.21)	1.30 [0.86, 1.96]	0.216
Living alone	1.42 (0.17)	4.15 [3.00, 5.74]	**<0.001**	0.95 (0.19)	2.57 [1.78, 3.73]	**<0.001**
UCLA‐LS	0.03 (0.01)	1.03 [1.02, 1.05]	**<0.001**	0.02 (0.01)	1.02 [1.00, 1.04]	**0.012**
NPI hallucination subscale score						
Female	1.17 (0.19)	3.24 [2.24, 4.68]	**<0.001**	1.11 (0.23)	3.05 [1.94, 4.80]	**<0.001**
Age	0.06 (0.01)	1.06 [1.03, 1.08]	**<0.001**	0.00 (0.02)	1.00 [0.97, 1.03]	0.919
Education duration	−0.22 (0.03)	0.80 [0.75, 0.85]	**<0.001**	−0.06 (0.04)	0.94 [0.87, 1.01]	0.103
Ever‐married status	0.20 (0.56)	1.22 [0.41, 3.67]	0.723	0.58 (0.63)	1.78 [0.52, 6.06]	0.358
Hearing impairment	1.39 (0.24)	4.02 [2.51, 6.44]	**<0.001**	0.91 (0.30)	2.48 [1.38, 4.47]	**0.002**
Use of psychotropic medications	1.11 (0.17)	3.02 [2.18, 4.18]	**<0.001**	0.95 (0.18)	2.58 [1.80, 3.71]	**<0.001**
MMSE	0.00 (0.03)	1.00 [0.95, 1.06]	0.964	0.03 (0.03)	1.03 [0.96, 1.10]	0.399
Mobility dependence	0.84 (0.18)	2.32 [1.63, 3.29]	**<0.001**	0.42 (0.22)	1.52 [0.98, 2.37]	0.062
Living alone	0.96 (0.17)	2.62 [1.87, 3.69]	**<0.001**	0.38 (0.21)	1.46 [0.97, 2.21]	0.073
UCLA‐LS	0.02 (0.01)	1.02 [1.00, 1.04]	**0.012**	0.02 (0.01)	1.02 [1.00, 1.04]	0.066

*Note*: Bold values indicate *p* < 0.05.

Abbreviations: *B*, unstandardized coefficient; CI, confidence interval; IRR, incidence rate ratio; MMSE, Mini‐Mental State Examination; SE, standard error; UCLA‐LS, the University of California, Los Angeles‐Loneliness Scale.

## DISCUSSION

In this study, we investigated the relationship between neuropsychiatric symptoms and psychosocial factors, specifically living alone and loneliness, in patients with MCI. Compared to patients living with family, those living alone exhibited significantly higher NPI scores for delusions, hallucinations, and disinhibition but significantly lower scores for apathy. Multivariate analysis revealed that depression and anxiety were linked to loneliness, while delusions were associated with both living alone and loneliness. On the other hand, hallucinations showed no significant associations with either living alone or loneliness.

Although loneliness is defined as the disparity between desired and actual interpersonal relationships,^25^ this study found no significant difference in loneliness scores between living‐alone and living‐with‐family groups. Previous research identifies contributing factors to loneliness, such as age, sex, bereavement of a spouse, lower education, poverty, and sensory impairments.^26^, ^27^ Loneliness scores were similar between the two groups despite the living‐alone group exhibiting older age, a higher proportion of women, lower education levels, and a lower rate of ever‐married status than the living‐with‐family group. This finding reinforces the idea that loneliness is a subjective experience influenced by complex factors. It also highlights the importance of independently examining living alone and loneliness as distinct concepts,^28^ particularly given their often conflated but fundamentally different nature, when analyzing their associations with neuropsychiatric symptoms.

Regarding affective symptoms in MCI, multivariate analysis demonstrated that loneliness was significantly associated with both depression and anxiety. Conversely, living alone showed no association with affective symptoms. These findings align with previous studies on older adults, which established strong bidirectional links between loneliness and affective symptoms.^29^, ^30^ Consistent with prior research,^31^ our results suggest that subjective loneliness has a stronger factor of association with affective symptoms than the objective state of living alone.

Delusions in MCI were associated with both living alone and loneliness, with a stronger association observed for living alone. These findings align with prior research on abnormal thinking and perception in MBI.^32^ However, studies in dementia have reported that delusions are primarily linked to loneliness rather than cohabitation status.^33^ These discrepancies may be partly explained by the degree of cognitive impairment, suggesting that when cognitive impairment is mild, living alone is more closely related to delusions than loneliness. In geriatric psychiatry, a relationship between delusions and solitary living has been suggested. In late paraphrenia, risk factors such as being female, single, socially isolated, experiencing hearing loss, having organic brain lesions, and exhibiting pre‐psychotic personality traits have been statistically identified.^34^ Janzarik's concept of “contact deficit paranoids”^35^ describes chronic psychosis in which a solitary living situation contributes to both the onset and amelioration of symptoms. In this context, delusions may function as a defense mechanism to mitigate loneliness by generating a “regeneration of lost interpersonal relationships.” A certain level of cognitive function may be necessary for such defense mechanisms to operate. Our findings that delusions in patients with MCI were associated with both living alone and loneliness may partially reflect these psychosocial risk factors described in late‐life psychosis.

For hallucinations, multivariate analysis showed no significant associations with either living alone or loneliness, whereas female sex, hearing impairment, and the use of psychotropic medications were significantly associated. Late‐life psychosis characterized by hallucinations and delusions has been reported to be more frequent in women, particularly those with hearing impairment and social isolation.^36^ This suggests that hearing impairment may have acted as a confounding factor for other variables, such as female sex and living alone, which were significant in the univariate analysis. A previous study reported a prevalence of auditory hallucinations of approximately 32.8% among older adults with hearing impairment.^37^ Consequently, our findings suggest that hearing impairments may be more important than living alone or loneliness in causing hallucinations in patients with MCI.

Sensitivity analyses that excluded hearing impairment and marital status demonstrated that the associations between living alone, loneliness, depression, anxiety, and delusions were largely consistent with the primary multivariate findings. This supports the stability of our main observations. In contrast, the results for hallucinations showed a slight discrepancy: the association with living alone only reached statistical significance in the sensitivity analysis. We suspect that hearing impairment, which was significantly associated with hallucinations in our primary multivariate model, may have acted as a confounding factor or limited the stability of the living arrangement estimates due to highly unbalanced distributions. It is important to note that the number of participants with hearing impairment was very small and that the assessment was limited to a qualitative check for impairments significant enough to interfere with the testing session, rather than quantitative audiometry. This limited precision in assessing sensory deficits likely introduced instability into the original model. Notably, in the original multivariate analysis, living alone already exhibited a trend towards an association with hallucinations (*p* = 0.073). Therefore, we believe that the results of both analyses are fundamentally consistent, which further supports the relationship between living arrangements and hallucinations after accounting for the influence of sensory‐related factors.

In the present study, female sex was associated with all the neuropsychiatric symptoms that were assessed. Previous studies have reported a higher prevalence of those neuropsychiatric symptoms in women.^38^, ^39^, ^40^ Our findings are consistent with these previous reports. Age was also associated with specific symptoms of neuropsychiatric symptoms in MCI. Depression and anxiety were more associated with younger age, while delusions were associated with older age. No significant association was observed between age and hallucinations. The inverse relationship between age and affective symptoms may reflect greater psychological distress among younger individuals with MCI. Previous studies have suggested that younger patients tend to have greater insight into cognitive decline, which can lead to more intense emotional responses, such as depression and anxiety.^41^ In contrast, delusions were positively associated with older age, which is consistent with previous reports indicating that psychotic symptoms in later life are closely linked to age‐related biological and psychosocial vulnerabilities.^20^ The absence of a significant association between age and hallucinations suggests that hallucinations in MCI may be less directly related to chronological age and more strongly influenced by specific factors. Previous studies have suggested that hallucinations in older adults may be associated with specific factors, such as female sex and sensory impairment, rather than with age itself.^36^ Overall, these findings suggest that the impact of age on neuropsychiatric symptoms in MCI varies. Affective symptoms tend to be more prevalent in younger individuals due to preserved insight and psychosocial stress, while delusions appear to reflect cumulative age‐related vulnerabilities.

Several limitations should be acknowledged. First, MCI was operationalized based on all cases meeting CDR = 0.5, without any stratification by etiology or biomarker‐based classification. This approach likely resulted in a sample that may include prodromal Alzheimer's disease, prodromal DLB, late‐life depression, and late‐life psychiatric disorders, as well as MCI, as these conditions are more heterogeneous. As these conditions have substantially different neuropsychiatric profiles, the lack of diagnostic differentiation limits the specificity of symptom‐related interpretations. In particular, the potential inclusion of individuals with prodromal Lewy body disease, which is often characterized by early hallucinations and other neuropsychiatric symptoms, may have influenced the findings related to hallucinations. Second, due to the cross‐sectional and retrospective design, causal relationships between neuropsychiatric symptoms, living alone, and loneliness cannot be inferred. Thus, the observed associations should be interpreted as correlational rather than indicative of psychosocial risk mechanisms. Third, several critical covariates were absent from our analysis, and even for the variables included, limitations in measurement precision remained. As this was a retrospective study based on existing clinical records, the range of available variables was limited by its very nature. Consequently, although multiple covariates were included, adjustment for several important potential confounders was incomplete. Specifically, information on detailed social characteristics, such as social network size, frequency of social contact, and availability of informal support, was not systematically recorded and therefore could not be included in the analysis. Furthermore, the variables that were included may have lacked sufficient granularity. While we included the mobility item of the PSMS as a partial proxy for physical health, this measure does not fully capture the broad impact of medical comorbidities. Living alone was defined as a binary variable measured at a single point in time. This approach does not consider the important factors such as the duration, voluntariness, or functional independence of the living arrangement, nor the availability of informal caregivers or emergency support systems. These dimensions are clinically relevant in geriatric psychiatry and may influence the development of affective and psychotic symptoms differently. Regarding marital history, the current marital status at the time of evaluation could not be fully captured, as the data included a broad history of divorce or bereavement. Hearing impairment was limited to a simple qualitative assessment only, and the small number of cases may have hindered our ability to detect its influence. Regarding the NPI ratings, although we only included data from informants who met the minimum frequency requirement for contact with patients living alone, the informants' cohabitation status may have influenced the findings. Specifically, even with frequent visits, non‐cohabiting caregivers have fewer opportunities for continuous observation than cohabiting caregivers. This difference in the “density” of observation could lead to variations in the detection and assessment of symptom severity, potentially resulting in an under‐ or overestimation of neuropsychiatric symptoms in the living‐alone group. Similarly, although loneliness was assessed as a subjective construct, it was not possible to explore the underlying social and contextual factors contributing to loneliness in detail. Future studies should move beyond binary classifications to capture these nuanced clinical dimensions. Finally, the presence of significant selection bias must be addressed. The study population consisted of patients attending a university hospital, which should have introduced referral bias and limited the external validity of the findings. This was evident in the demographic profile of our cohort. For example, the prevalence of having been married was nonetheless very high in both groups. Additionally, the proportion of patients with a history of psychiatric care was extremely low in both groups. This setting may disproportionately include individuals with higher educational attainment, preserved insight, and greater help‐seeking behavior, thereby limiting the generalizability of the findings to community‐dwelling populations with MCI.

Despite these limitations, this study benefits from a relatively large sample size and the parallel investigation of social isolation and loneliness. Our study demonstrated that the associations of living arrangements and loneliness with neuropsychiatric symptoms in patients with MCI vary depending on the specific symptom domain. Affective symptoms were significantly associated with loneliness, while delusions were associated with both living alone and loneliness. In contrast, hallucinations showed no significant association with either factor. These findings suggest that the impact of objective solitary living and subjective loneliness is symptom‐specific, and consequently, the effectiveness of interventions targeting these social factors may differ across various neuropsychiatric symptoms. Future longitudinal studies across various disease etiologies are necessary to establish the causal relationships between living arrangements, loneliness, and neuropsychiatric symptom progression in MCI, thereby refining clinical approaches.

## AUTHOR CONTRIBUTIONS

**Kayo Takeda**: Conceptualization; data curation; resources; formal analysis; writing—original draft. **Hideki Kanemoto**: Conceptualization; methodology; funding acquisition; project administration; supervision; writing—review and editing. **Takashi Suehiro**: Conceptualization; writing—review and editing. **Yuto Satake**: Data curation; resources. **Daiki Taomoto**: Data curation; resources. **Shigeki Katakami**: Data curation; resources. **Kenji Yoshiyama**: Project administration. **Manabu Ikeda**: Project administration; supervision; writing—review and editing.

## CONFLICT OF INTEREST STATEMENT

The authors declare no conflicts of interest.

## ETHICS APPROVAL STATEMENT

This study received approval from the Research Ethical Committee of the University of Osaka Hospital (Suita, Japan, approval number 19117).

## PATIENT CONSENT STATEMENT

The study was approved by the institutional review board, and patient data were retrospectively collected through an opt‐out consent process.

## CLINICAL TRIAL REGISTRATION

N/A (retrospective observational study).

## Supporting information

Supporting Information.

## Data Availability

The data that support the findings of this study are available on request from the corresponding author. The data are not publicly available due to privacy or ethical restrictions.
