# Supplementary material for: Associations of living alone and loneliness with neuropsychiatric symptoms in individuals with mild cognitive impairment: A retrospective cross‐sectional study
Source: PCN Rep. 2026 Feb 10;5(1):e70299. doi: 10.1002/pcn5.70299 (PMC12887818; doi:10.1002/pcn5.70299)
Supplement: Supplementary file 1 — Supporting Information. [file PCN5-5-e70299-s001.docx]

Supplementary Table 1. Sensitivity analysis of negative binomial regression models excluding hearing impairment and marital status

| NPI depression subscale score | *B* (SE) | IRR [95% CI] | *p* |
| --- | --- | --- | --- |
| Female | 0.99 (0.21) | 2.70 [1.78, 4.11] | **<0.001** |
| Age | -0.03 (0.01) | 0.97 [0.94, 0.99] | **0.009** |
| Education duration | 0.16 (0.03) | 1.17 [1.10, 1.25] | **<0.001** |
| Use of psychotropic medications | 0.72 (0.18) | 2.06 [1.46, 2.90] | **<0.001** |
| MMSE | 0.00 (0.03) | 1.00 [0.95, 1.06] | 0.876 |
| Mobility dependence | -0.03 (0.22) | 0.97 [0.64, 1.48] | 0.895 |
| Living alone | 0.17 (0.20) | 1.19 [0.80, 1.77] | 0.391 |
| UCLA-LS | 0.05 (0.01) | 1.05 [1.03, 1.07] | **<0.001** |
| NPI anxiety subscale score | *B* (SE) | IRR [95% CI] | *p* |
| Female | 0.45 (0.20) | 1.57 [1.05, 2.34] | **0.027** |
| Age | -0.03 (0.01) | 0.97 [0.95, 1.00] | **0.035** |
| Education duration | -0.03 (0.03) | 0.97 [0.91, 1.04] | 0.407 |
| Use of psychotropic medications | 0.75 (0.17) | 2.12 [1.52, 2.95] | **<0.001** |
| MMSE | -0.01 (0.03) | 0.99 [0.94, 1.04] | 0.645 |
| Mobility dependence | -0.22 (0.21) | 0.80 [0.53, 1.22] | 0.297 |
| Living alone | 0.24 (0.20) | 1.27 [0.87, 1.87] | 0.221 |
| UCLA-LS | 0.02 (0.01) | 1.02 [1.00, 1.04] | **0.012** |
| NPI delusions subscale score | *B* (SE) | IRR [95% CI] | *p* |
| Female | 1.02 (0.20) | 2.77 [1.86, 4.14] | **<0.001** |
| Age | 0.03 (0.01) | 1.03 [1.01, 1.06] | **0.016** |
| Education duration | -0.07 (0.04) | 0.93 [0.87, 1.00] | **0.049** |
| Use of psychotropic medications | 0.26 (0.17) | 1.30 [0.93, 1.82] | 0.128 |
| MMSE | 0.00 (0.03) | 1.00 [0.95, 1.06] | 0.889 |
| Mobility dependence | 0.27 (0.21) | 1.31 [0.87, 1.98] | 0.191 |
| Living alone | 1.01 (0.18) | 2.75 [1.93, 3.91] | **<0.001** |
| UCLA-LS | 0.02 (0.01) | 1.02 [1.00, 1.04] | **0.015** |
| NPI hallucination subscale score | *B* (SE) | IRR [95% CI] | *p* |
| Female | 0.02 (0.01) | 1.02 [1.00, 1.04] | **0.015** |
| Age | 0.02 (0.01) | 1.02 [0.99, 1.05] | 0.135 |
| Education duration | -0.09 (0.04) | 0.91 [0.85, 0.98] | **0.015** |
| Use of psychotropic medications | 0.93 (0.18) | 2.54 [1.78, 3.62] | **<0.001** |
| MMSE | 0.03 (0.03) | 1.03 [0.96, 1.10] | 0.390 |
| Mobility dependence | 0.40 (0.22) | 1.50 [0.97, 2.32] | 0.068 |
| Living alone | 0.45 (0.20) | 1.56 [1.05, 2.31] | **0.026** |
| UCLA-LS | 0.02 (0.01) | 1.02 [1.00, 1.04] | 0.067 |

Note: **Bold values** indicate *p* <0.05.
Abbreviations: NPI, Neuropsychiatric Inventory; B, unstandardized coefficient; SE, standard error; IRR, incidence rate ratio; CI, confidence interval; MMSE, Mini-Mental State Examination; UCLA-LS, the University of California, Los Angeles Loneliness Scale
